# Cotransplantation of Mesenchymal Stem Cells and Immature Dendritic Cells Potentiates the Blood Glucose Control of Islet Allografts

**DOI:** 10.1155/2017/4107943

**Published:** 2017-12-19

**Authors:** Guanghui Long, Guangtao Zhang, Fangting Zhang, Minghua Li, Dongshuo Ye, Dengke Yang, Yinke Yang

**Affiliations:** ^1^Department of Hepatobiliary Surgery, Peking University Shenzhen Hospital, Shenzhen, China; ^2^Center Laboratory, Peking University Shenzhen Hospital, Shenzhen, China; ^3^Shenzhen BioScien Pharmaceuticals Co. LTD, Shenzhen, China

## Abstract

**Background:**

Transplantation of islets is a promising alternative to treat type 1 diabetes (T1D), but graft rejection is the major obstacle to its application in clinical practice. We evaluated the effects of mesenchymal stem cells (MSCs) and immature dendritic cells (imDCs) on islet transplantation in diabetic model.

**Methods:**

The streptozotocin T1D model was established in BABL/c mice. Rat islets were isolated and identified with dithizone (DTZ) staining. MSCs and imDCs were isolated from bone marrow of syngenic mice. Islets, alone or along with MSCs and/or imDCs, were transplanted to the left kidney capsule of diabetic mice. The blood glucose levels and glycosylated hemoglobin levels after transplantation were monitored.

**Results:**

Cotransplantation significantly decreased blood glucose and glycosylated hemoglobin levels in the diabetes mice. Transplantation of 200 islets + 2 × 10^5^ MSCs + 2 × 10^5^ imDCs could not only restore normal blood glucose levels, but also significantly prolong graft survival for 12.6 ± 3.48 days.

**Conclusions:**

Cotransplantation of allogenic islets with imDCs and/or MSCs can significantly promote graft survival, reverse hyperglycemia, and effectively control the glycosylated hemoglobin levels.

## 1. Introduction

According to the World Health Organization, there were 382 million diabetic patients in the world in 2013 and diabetes should become the seventh leading cause of death by 2030 [1]. Different from type 2 diabetes, type 1 diabetes (T1D) is recognized as an autoimmune disorder in which the insulin-producing *β*-cells in the Langerhans islets within the pancreas are the target of T cell-mediated destruction [2, 3], resulting in lifelong dependence upon exogenous insulin [4]. Only approximately 30% of T1D individuals meet the American Diabetes Association goal of hemoglobin A1c (HbA1c) level of 7.0% (53 mmol/mol) for adults and 7.5% for children, indicating the need for better approaches to diabetes management [5].

Since the first successful allogeneic pancreatic fragment transplantation in patients with T1D in 1980, recent clinical trials indicated that islet transplantation is a promising treatment for T1D. An international trial based on the Edmonton protocol yielded 58% insulin independence at 1 year after operation [6]. A recent phase 3 trial of human islets transplantation in T1D patients complicated by severe hypoglycemia showed exciting results: 87.5% of the subjects at 1 year and 71% at 2 years achieved HbA1c < 7.0% [7]. Nevertheless, in addition to the quality and quantity of donor islets, revascularization of graft islets after transplantation [8] and immune rejection [3] are critically affecting the success rate.

Studies have shown that mesenchymal stem cells (MSCs) and islet cotransplantation promoted graft revascularization and function in diabetic animals [9–11], possibly through their immunosuppressive activity on proinflammatory T cells [12] and inhibition of dendritic cell (DC) maturation [13]. In addition, immature dendritic cells (imDCs) can prolong the survival of islet xenograft by inhibiting the activation of T cells [14] and the development of alloantigen-specific CD4^+^ CD25^+^ Treg cells [15].

In the present study, we hypothesized that MSCs and imDCs cooperatively inhibit transplant rejection and therefore transplanted rat pancreatic islets along with mouse MSCs and/or imDCs to diabetic mice. We therefore evaluated whether this combinational transplantation could benefit long-term blood glucose control. The data suggest that the transplantation of pancreatic islets together with MSCs and imDCs was superior for blood glucose control compared to the transplantation of pancreatic islets alone, or pancreatic islets with either MSCs or imDCs.

## 2. Materials and Methods

### 2.1. Materials

BALB/c mice (6 weeks old) and SD rats (250–270 g, 6–8 weeks old) were purchased from Guangdong Medical Experimental Animal Center and maintained according to “Regulations for the administration of affairs concerning experimental animals” of the People's Republic of China. Fetal bovine serum, RPMI-1640, DMEM/F12 medium, HBSS, penicillin, and streptomycin were purchased from HYCLONE (Logan, Utah, USA). MTT and DMSO were purchased from Shanghai Sangon Biotech Co., Ltd. (Shanghai, China). Lymphocyte separation medium (Histopaque, 1.077 g/mL), collagenase V, streptozotocin (STZ), dithizone (DTZ), and acridine orange/propidium iodide (AO/PI) were purchased from SIGMA (San Francisco, USA). Rat INS (insulin) ELISA Kit was purchased from Elabscience (Wuhan Eli Reiter Biotechnology Co., Ltd., Wuhan, China). Mouse glycosylated hemoglobin (HbA1c) ELISA Kit was purchased from Shanghai Yanjin Biotech Co., Ltd. (Shanghai, China). Anti-mouse CD11c-FITC, anti-mouse CD86-FITC, anti-mouse CD80-FITC, anti-mouse MHC-II-FITC, rat IgG2b K isotype-FITC, Armenian hamster IgG isotype-FITC, and rat IgG2a K isotype-FITC were purchased from eBioscience (San Diego, USA). Mouse recombinant granulocyte-macrophage colony stimulating factor (rmGM-CSF) and recombinant mouse interleukin-4 (rmIL-4) were purchased from PEPROTECH (New Jersey, USA). Blood glucose test strips and ACCU-CHEK Performa glucose meter were from Roche (Basel, Switzerland).

### 2.2. Isolation and Identification of Bone Marrow Mesenchymal Stem Cells

The isolation and culture of MSCs were performed as described previously [16]. Briefly, 6–8-week-old male BALB/c mice were sacrificed by cervical dislocation. The bilateral femurs and tibias were dissected and the joints were cut off. The bone cavities were flushed with complete DMEM/F12 medium until the bones became pale. The eluent was filtered through a 200-mesh filter, and the filtrate was collected by centrifugation. The cell pellets were resuspended, adjusted to 2–5 × 10^6^/mL, and seeded in a 6-well plate. The plate was incubated at 37°C, in a 5% CO_2_ incubator. Three hours later, the medium was refreshed to remove nonadherent cells. Thereafter, the medium was changed every 8 hours for the first 72 hours after isolation. The cells were digested with 0.25% trypsin and passaged when they reached 90% confluence.

The MSCs at passage 3 were seeded in a 6-well plate at 2 × 10^4^/well. For osteogenic induction, the medium was changed with osteogenic induction medium (DMEM/F12 containing 10^−7^ mol/L dexamethasone, 10 mmol/L beta-sodium glycerol phosphate, 0.2 mmol/L ascorbic acid, and 10% FBS) when the cells grew to 80–90% confluence. Two weeks after induction, the cells were stained with Alizarin red dye or Von Kossa's staining. For adipogenic induction, the confluent cells were incubated with 2 mL adipogenic solution A (DMEM-LG containing 10^−6^ mol/L dexamethasone, 0.5 mmol/L IBMX, 0.2 mmol/L indomethacin zinc, 10 *μ*g/mL insulin, and 10% FBS). Three days later the medium was replaced with adipogenic solution B (DMEM-LG containing 10 *μ*g/mL insulin and 10% FBS) for 24 h. The induction included three cycles of alternate incubation of adipogenic solution A (3 days) and solution B (24 h). The lipid droplets were fixed with 10% neutral formaldehyde for 10 min, stained with oil red for 30 min, and observed under an inverted microscope.

### 2.3. Isolation and Identification of Immature Dendritic Cells

The imDCs were isolated as described previously [17]. The cells were isolated from femurs and tibias following the same procedures as for MSC isolation. The collected cells were incubated with 1 mL erythrocyte lysis buffer (Shanghai Sangon Biotech Co., Ltd.) for 30–60 s with gentle tapping and resuspended with RMPI-1640 medium containing 10% FBS and 1% penicillin/streptomycin after centrifugation. The isolated cells were seeded in 10-cm culture dishes at 2 × 10^6^/mL. The medium was supplemented with 20 ng/mL recombinant mouse GM-CSF and 10 ng/mL recombinant mouse IL-4. On days 3 and 5 the medium was semirefreshed with fresh medium containing GM-CSF and IL-4. On day 7, the cells were stained with CD11c, MHC-II, CD86, and CD80 antibodies and identified with flow cytometry (BD, Franklin lakes, USA). The same procedures were performed to isolate total DCs from SD rats.

### 2.4. Purification and Identification of Rat Islets

Primary islet isolation was performed according to a published method [18]. The adult SD rats (8–10 weeks old, weighing 250–300 g) were anesthetized with sodium pentobarbital. The pancreatic main duct was ligated and 8–10 mL collagenase V (1 mg/mL) was injected into the pancreas via the common bile duct. Then the pancreas was removed and incubated at 37.5°C for 20 min. The digestion was stopped by Hanks solution containing 10% FBS and the cells were filtered through a 40-mesh filter. Islet cells were purified with Histopaque-1077, stained with 5 mL of dithizone, and observed using light microscopy. Counting and statistical analysis of islet equivalents purity were repeated three times. Islet yield (expressed as islet equivalents, IEQ) and purity were determined according to standard methods [19]. Islet viability was assessed with acridine orange (AO) and propidium iodide (PI) staining [20].

### 2.5. Glucose-Stimulated Insulin Secretion by Islet Cells

This assay was conducted according to published methods [21]. The stimulation index (SI) = insulin concentration in 16.8 mM glucose/insulin concentration in 2.8 mM glucose.

### 2.6. Mixed Lymphocyte Reaction (MLR) and Lymphocyte Proliferation Assay

T lymphocytes isolated from the peripheral blood of Sprague-Dawley rats were used as the reaction cells (R). The mice MSCs, imDCs, or imDC + MSCs were used as the stimulator cells (S) after they were treated with 40 *μ*g/mL of mitomycin C for 1 h. The cells were mixed at a R : S ratio of 10 : 1, resuspended in 200 *μ*L of RPMI-1640 supplemented with 10% fetal bovine serum (FBS), and seeded into a 96-well plate. The MLR assay included five groups: group A (negative control), made of rats total DCs mixed with T cells (S : R = 1 : 10, i.e., 1 × 10^4^ of DCs and 1 × 10^5^ of T cells for each well); group B (positive control), made of mDCs (imDCs stimulated with 10 ng/ml of LPS for 24 h) mixed with T cells (S : R = 1 : 10); group C, made of MSCs and T cells (S : R = 1 : 10); group D, made of imDCs and T cells (S : R = 1 : 10); and group E, made of a mixture of imDCs, T cells, and MSCs (1 × 10^5^ T cells, 5 × 10^3^ MSCs, and 5 × 10^3^ imDCs in each well). All the cells were incubated at 37°C, in a 5% CO_2_ incubator for 3 days. T cell proliferation was analyzed with the MTT assay. Lymphocyte activation stimulation index (LASI) = experimental group OD_570_/control group OD_570_.

### 2.7. Establishment of the Diabetic Mice Model

Streptozotocin (STZ) solution was prepared immediately before use. STZ was dissolved in 0.1 M sodium citrate buffer (pH 4.5) at 12 mg/mL and filtered with a 0.22-*μ*m filter. Male BALB/c mice (8–10 weeks old) were maintained under specific pathogen-free conditions. The experiments were performed after one week of acclimation. The mice were injected intraperitoneally with the STZ solution at 150 mg/kg. The fasting blood glucose was measured 72 h after injection and for 5 consecutive days thereafter. Mice with a daily glucose concentration > 16.7 mM were considered diabetic [22].

### 2.8. Animal Grouping and Detection of Blood Glucose Levels and Glycated Hemoglobin Levels

Diabetic BALB/c mice were randomly divided into five groups receiving islets and syngeneic mouse imDCs and/or MSCs transplantation. After intraperitoneal 2% sodium pentobarbital anesthesia (60 mg/kg), a small incision was made in the back to expose the left kidney. Group A (*n* = 5): diabetic control group injected with saline. Group B (*n* = 6): 200 islets were suspended in 0.1 ml saline and transplanted to the left kidney capsule. Group C (*n* = 6): 200 islets and 2 × 10^5^ MSCs were transplanted to the left renal capsule. Group D (*n* = 6): 200 islets and 2 × 10^5^ imDCs were transplanted to the left kidney capsule. Group E (*n* = 5): 200 islets, 2 × 10^5^ MSCs, and 2 × 10^5^ imDCs were cotransplanted to the left renal capsule.

Before transplantation, and every 24 h after transplantation, tail vein blood was sampled to detect glucose level. Blood glucose levels > 11.1 mM on two consecutive days after transplantation were defined as transplant rejection, while blood glucose levels > 16.7 mM on two consecutive days was defined as graft loss.

After transplantation, orbital blood was sampled every four days and the levels of glycated hemoglobin (HbA1c) were determined by ELISA. The concentration of glycated hemoglobin was expressed as % HbA1c = HbA1c (g/dl)/Hb (g/dl) × 100%.

### 2.9. Statistical Analysis

Statistical analysis was performed using SPSS 13.0 for Windows (SPSS Inc., Chicago, USA). Data were expressed as mean ± standard deviation and the difference among groups was analyzed using analysis of variance (ANOVA). *p* < 0.05 indicated a statistically significant difference.

## 3. Results

### 3.1. Characterization of Isolated MSCs

**Figure 1:**
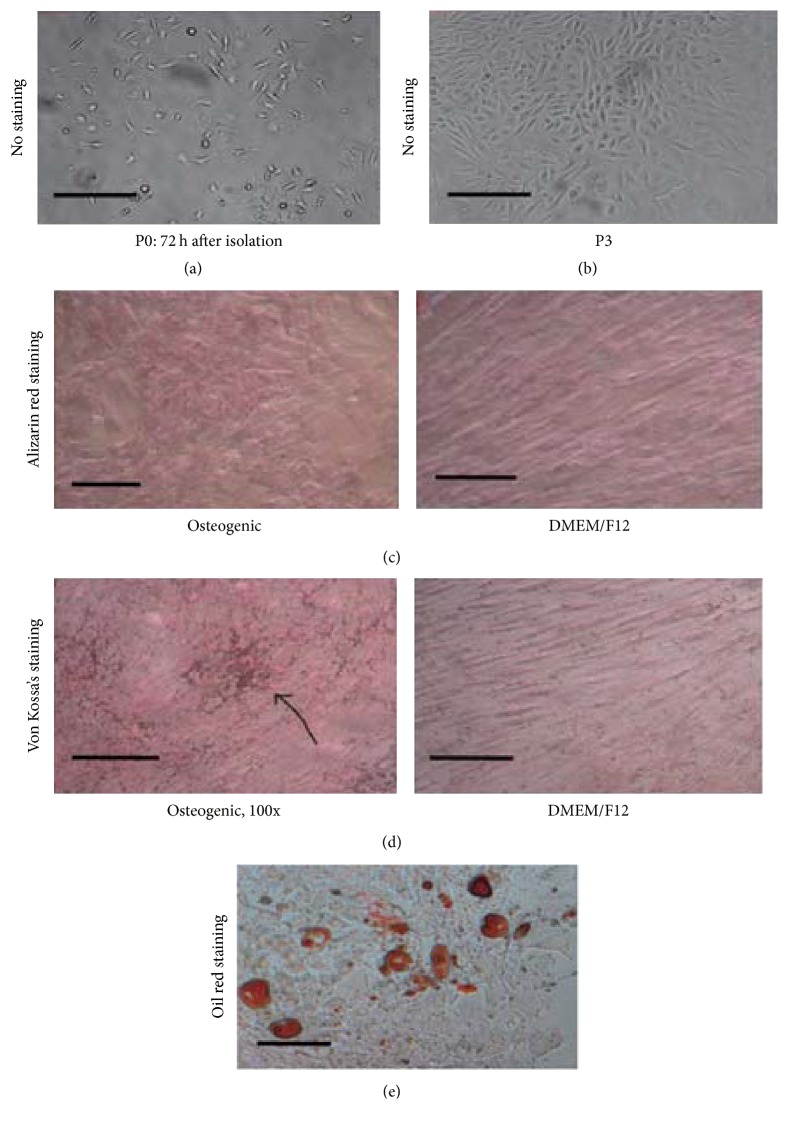
*Morphology and osteogenic induction of mouse mesenchymal stem cells*. Isolated MSCs attached to the culture dish at passage 0 (a) and showed uniform morphology at passage 3 (b). Alizarin red staining shows calcium nodules in the cytoplasm (c) and Von Kossa stain reveals mineral deposition (d) in MSCs. After 2 weeks of adipogenic induction, the majority of cells showed lipid droplets (e). P3: passage 3; magnification ×100; Scale bar = 100 *μ*m.

After 72 h of adherent culture, the morphology of the mouse bone marrow cells was observed under the microscope. The morphologies of the cells were various (Figure 1(a)), but after 1 week of culture, most of the cells were spindle-like and the morphology became uniform after repeated subculture (Figure 1(b) shows the cells at passage 3). We performed osteogenesis induction with the mouse bone marrow cells at passage 3. Alizarin red staining showed the formation of calcium nodules in the cytoplasm (Figure 1(c)). Von Kossa stain revealed mineral deposition in the bone located in the island-shaped cell aggregation (Figure 1(d)). After two weeks of adipogenic induction, the majority of cells showed lipid droplets (Figure 1(e)), suggesting that the isolated cells were MSCs.

### 3.2. Identification of imDCs

**Figure 2:**
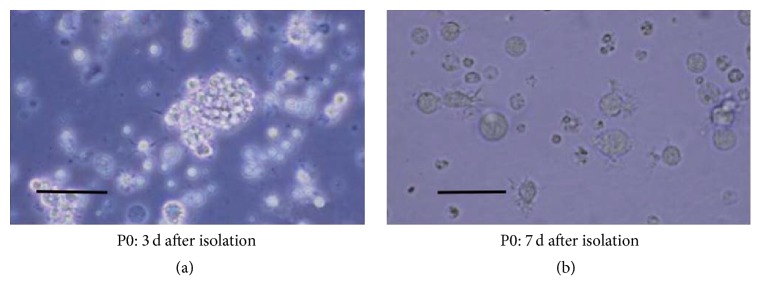
*Morphology of immature dendritic cells* (magnification ×100; Scale bar = 100 *μ*m.). Mouse bone marrow cells were induced in medium containing GM-CSF and IL-4 for 3 h and only the adherent cells were retained. (a) After 3 days of culture, a large number of colonies were formed. (b) Seven days later, the morphology of cells became irregular, with some irregular spike-like projections on the cell surface.

**Figure 3:**
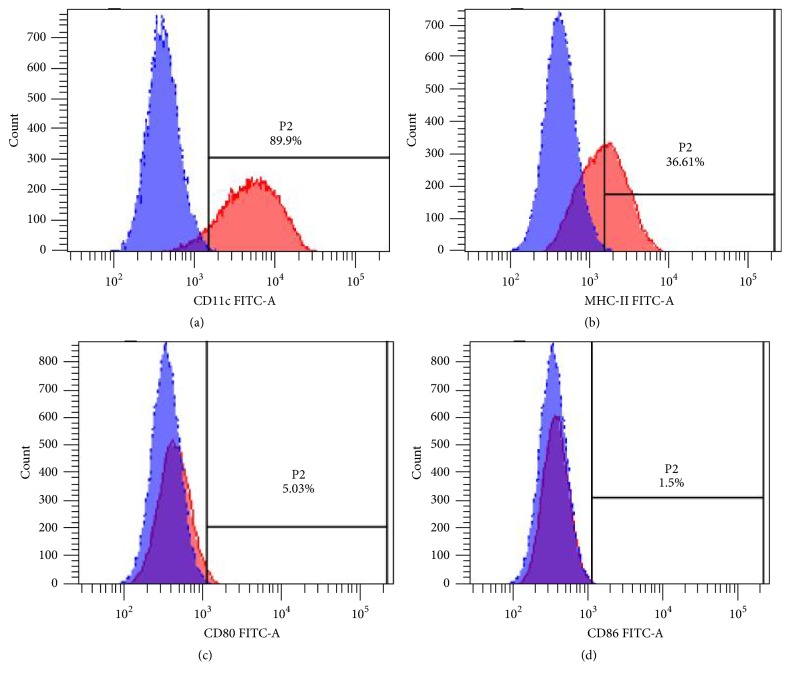
*Flow cytometry analysis of imDC surface markers*. On day 7 after stimulation, the cells were stained with CD11c, MHC-II, CD86, and CD80 antibodies and identified with flow cytometry: 89.9% of the cells were CD11c-positive (a) and 36.61% were MHC-II positive (b), while only 5.03% of cells were CD80-positive (c) and 1.5% cells were CD86-positive (d), which are consistent with the surface characteristics of imDCs.

Mouse bone marrow cells were induced in medium containing GM-CSF and IL-4 for 3 h and only the adherent cells were retained. After 3 days of culture, a large number of colonies were formed (Figure 2(a)). The size and morphology changed with prolonged incubation time. Seven days later, the morphology of cells became irregular, with some irregular spike-like projections on the cell surface (Figure 2(b)). Flow cytometry showed that 89.9% of the cells were CD11c-positive (Figure 3(a)) and 36.61% were MHC-II positive (Figure 3(b)), while only 5.03% of cells were CD80-positive (Figure 3(c)) and 1.5% cells were CD86-positive (Figure 3(d)), which is consistent with the surface characteristics of imDCs.

### 3.3. Morphology and Viability of Purified Islet Cells

**Figure 4:**
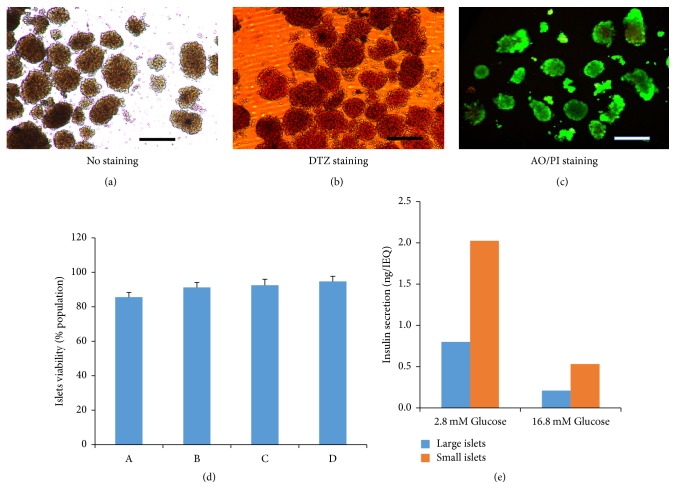
*Morphological observation and cell viability of rat islets*. (a) Morphology of the purified islets. (b) DTZ staining showing that 67.0 ± 9.9% of the cells were DTZ-positive. (c) AO/PI (acridine orange/propidium iodide) staining showing the live cells (in green). Scale bar = 100 *μ*m. (d) Islet viability in four independent isolations.* (e) Glucose-stimulated release of insulin*. The islets with a diameter of >150 *μ*m were termed as large islet, while <150 *μ*m were classified as small islets. To test the secretion of insulin by the isolated islets, the islets were incubated with different concentrations of glucose (16.8 mM and 2.8 mM) and the insulin levels were detected by ELISA.

The morphology of purified islets is shown in Figure 4(a). The islet cells were scarlet with DTZ staining (Figure 4(b)), while exocrine cells were devoid of staining. The DTZ-positive cells accounted for 67.0 ± 9.85%. When the purified islets were stained with AO/PI, the living cells were stained green, while the dead cells were red (Figure 4(c)). We randomly analyzed the viability of pancreatic islets from four rats and found that the islet viability after purification was 91 ± 3% (Figure 4(d)). These results show that we had purified islets with high purity and viability.

### 3.4. Glucose-Stimulated Insulin Release from Islets

To test the secretion of insulin by the isolated islets, the islets were incubated with different concentrations of glucose and the insulin levels were detected by ELISA. As shown in Figure 4(e), under 16.8 mM and 2.8 mM glucose stimulation, the insulin levels of large islets were 2.05 ng/IEQ and 0.82 ng/IEQ, respectively, and insulin levels secreted by small islets were 0.52 ng/IEQ and 0.21 ng/IEQ, respectively. This indicates that the isolated rat islets in culture still have a good ability to secrete insulin under the stimulation of glucose. The average SI was 2.53 ± 0.29.

### 3.5. imDCs and MSCs Inhibited the Proliferation of T Lymphocytes In Vitro

**Figure 5:**
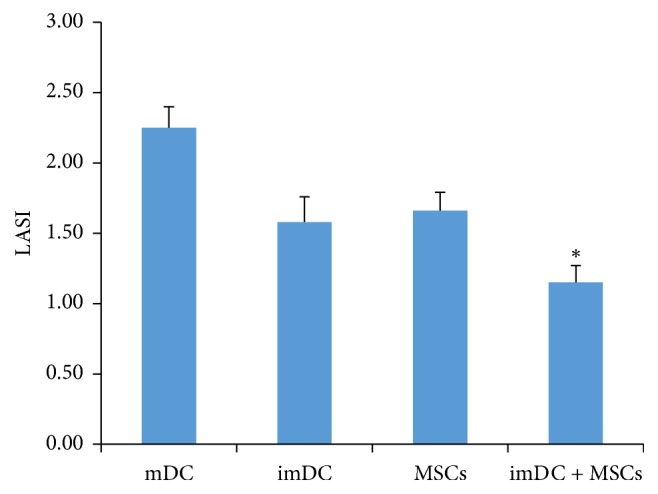
*Activation of T cells stimulated by imDCs or MSCs, as determined by the MTT assay*. To investigate the inhibitory effects of imDCs and/or MSCs on T cells, we conducted a MLR assay to compare the proliferation of T cells in different coculture groups. Lymphocyte activation stimulation index (LASI) = experimental group OD_570_/control group OD_570_. Statistical method: one-way ANOVA. ^*∗*^*p* < 0.05 compared with the mDC group.

To investigate the inhibitory effects of imDCs and/or MSCs on T cells, we conducted a MLR assay to compare the proliferation of T cells in different coculture groups. We found that either imDCs or MSCs could inhibit the proliferation of T cells (SI = 1.58 ± 0.18 for imDCs-T cells and SI = 1.66 ± 0.13 for MSCs-T cells) in the in vitro coculture system and that imDCs + MSCs + T cell significantly inhibited T cell stimulation (SI = 1.15 ± 0.12, *p* < 0.05) (Figure 5) compared to the other groups, indicating that imDCs + MSCs had a pronounced inhibitory effect on T cell proliferation.

### 3.6. Detection of Blood Glucose Levels and Glycated Hemoglobin after Transplantation

**Figure 6:**
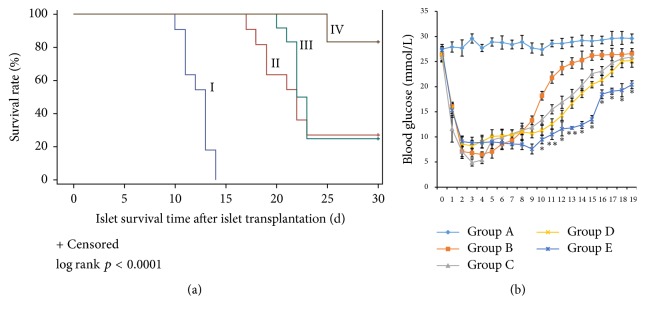
(a) Islets survival rate after islet transplantation with different cells. I: 200 Islets; II: 200 Islets + 2 × 10^5^ MSCs; III: 200 Islets + 2 × 10^5^ imDC; VI: 200 Islets + 2 × 10^5^ MSCs + 2 × 10^5^ imDC. Islet graft alone (I) was all rejected within 14 days with mean survival time (MST) of 12.27 ± 0.41 days. Islets + MSCs (II) and Islets + imDC (III) achieved prolonged survival, with 20.91%  ± 0.69 and 22.25%  ± 0.28, respectively. Islets + MSCs + imDC (IV) had the highest survival rate of 83.33 (*p* < 0.01). (b) Daily blood glucose levels after islets transplantation in mice. Statistical method: one-way ANOVA. ^*∗*^*p* < 0.05, ^*∗∗*^*p* < 0.01 compared with the control group.

Compared with the control group, the blood glucose levels of the diabetic mice transplanted with 200 islets alone decreased sharply in the first two days after transplantation but gradually increased from day 5, becoming >11.1 mM on day 7 and >16.7 mM on day 10. The mice transplanted with 200 islets and 2 × 10^5^ MSCs maintained low levels of blood glucose (<11.1 mM) for a longer duration (8.0 ± 2.3 d) and the blood glucose levels were <16.7 mM until day 14 after transplantation. The mice transplanted with 200 islets and 2 × 10^5^ imDCs also maintained low levels of blood glucose for a long time (7.0 ± 2.7 d). The blood glucose levels in the mice transplanted with 200 islets, 2 × 10^5^ MSCs, and 2 × 10^5^ imDCs maintained low levels of blood glucose for an even longer duration (12.6 ± 3.5 d), and, on day 7, the blood glucose levels were lower than in the other groups (Figure 6). In summary, the results showed that cotransplantation of these three cell populations could potentiate blood glucose control of transplanted islets.

**Figure 7:**
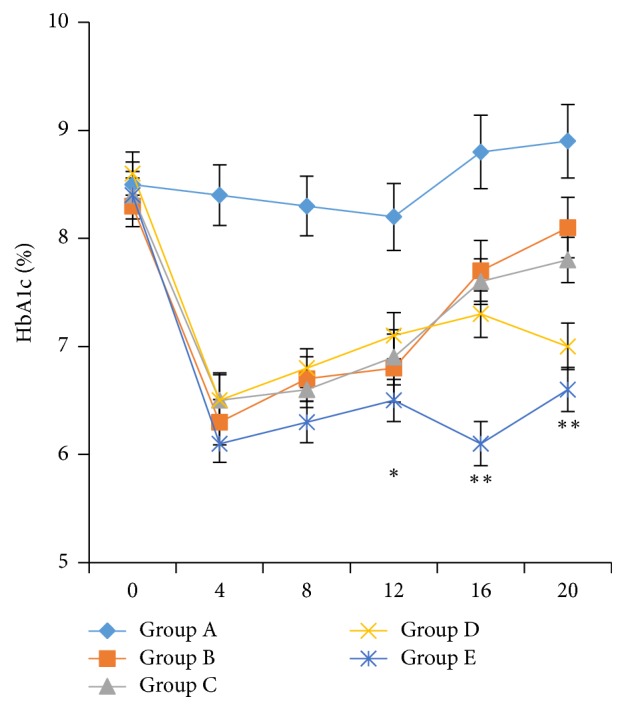
*HbA1c levels after islets transplantation in mice*. Statistical method: one-way ANOVA. ^*∗*^*p* < 0.05, ^*∗∗*^*p* < 0.01 compared with the control group.

Consistent with the results of blood glucose, the glycated hemoglobin levels in each transplantation group were well controlled within 12 days after transplantation, compared with the control group (*p* < 0.05), indicating that islet transplantation can effectively control blood glucose levels in diabetic mice. Nevertheless, the HbA1c levels increased over time. On day 20 after transplantation, the HbA1c levels were 8.1 ± 0.56% in group B (islets), indicating that glycemic control was not ideal. The HbA1c levels in group D (islets + MSCs + imDCs) were 7.3 ± 0.43% (*p* < 0.05) on day 16 and 7.0 ± 0.43% (*p* < 0.05) on day 20, suggesting that glycemic control of group D was better than in groups B and C. The HbA1c levels in group E were controlled to <6.5% (*p* < 0.01) for the first 16 days, and the HbA1c value was 6.6 ± 0.41% on day 20 (Figure 7). Taken together, cotransplantation of islets + MSCs + imDCs resulted in a better glycemic control than islets alone or cotransplanted with either MSCs or imDCs.

## 4. Discussion

In this study, we cotransplanted isolated islet cells along with MSCs and imDCs to explore whether MSCs and imDCs could contribute to a better glucose control in vivo. The results showed that cotransplantation of rat islets with mouse MSCs plus imDCs significantly enhanced islet grafts to reverse hyperglycemia (indicated by lower blood glucose and HbA1c levels) in mice with T1D.

To prevent immune rejection of islets after transplantation, transplant recipients require lifelong delivery of immunosuppressive drugs to maintain tolerance, which are usually associated with adverse effects [23]. DCs with tolerant characteristics are attracting much attention because they play important roles in maintaining immune homeostasis. Among various subpopulations of DCs, imDCs induce immune tolerance and improve long-term survival of allografts in the absence of immunosuppressive agents, which is considered to be an effective alternative to inhibit transplant rejection [24, 25]. Nevertheless, the immature phenotype of tolerant DCs may be unstable, as they become mature when they encounter a secondary stimulus and then act as antigen-presenting cells to initiate immune rejection [26]. To render DCs maturation-resistance, imDCs were fixed by resuspension in 2% paraformaldehyde [27]. It has been reported that the fixed imDCs extended the islets survival to 120 days after cotransplantation but the fresh imDCs did not contribute to graft survival (failed within 20 days after cotransplantation) [27]. This underlined that the immaturity of DCs is critical for allograft survival. In our study, when the islet cells and imDCs were transplanted together to diabetic mice, both the blood glucose levels and HbA1c could be maintained at a low level for longer time compared with islets alone (Figure 7), possibly confirming that an immune tolerance was induced by imDCs. The imDCs stimulated with GM-CSF and IL-4 are different from the fresh imDCs [27] and may contribute to longer survival of transplanted islets. On the other hand, the imDCs may become mature in vivo and this may be why we did not observe a longer normoglycemia.

MSCs are another cell population that can alleviate transplant rejection through their immunosuppressive effects on various immune cells and benefits for the residence of allogenic islets. Cotransplantation of MSCs with pancreatic islets improved islet graft function by promoting graft vascularization [28] and inhibiting immune rejection. In a model of allogenic pancreatic islet transplantation, the administration of MSCs resulted in the prolonged survival of islets and led to long-term stable normoglycemia [27]. In this study, MSCs were colocalized at the graft site where they blocked the activation and expansion of alloreactive T cells. Gao et al. found that transplanted MSCs in diabetic animals promoted the regeneration and repair of *β* cells [10]. It was also reported that MSCs alleviated rejection through their suppressive effects through T lymphocyte subsets and DCs when MSCs was cotransplanted with islets into the kidney capsules of diabetic C57LB/6 mouse. The numbers of T helper type 1 (Th1), naïve T cells, and memory T cells in peripheral blood decreased after transplantation. In addition, the maturation, endocytosis, and IL-12 secretion of DCs in recipient mice were suppressed [29]. Therefore, we hypothesized that MSCs maintain immature phenotype of imDCs and this interaction in turn enhances the suppression of transplant rejection against allogenic islets. Herein, we confirmed that bone marrow-derived MSCs prolonged the survival of mixed transplantation graft in order to maintain low blood glucose levels. In addition, MSCs and imDCs seemed to have synergetic effects blood glucose control after transplantation (Figure 5). Nevertheless, the interaction between MSCs and imDCs in vivo after cotransplantation is still largely unknown. Whether MSCs and imDCs work cooperatively or independently to induce immunosuppression, or even counteract with each other directly or indirectly, is still an open question.

## 5. Conclusions

In conclusion, we evaluated a novel cotransplantation of pancreatic islets with MSCs and imDCs in diabetic mice. More effective and significantly prolonged blood glucose control was achieved in this cotransplantation of three different cell populations compared with islets cotransplanted with either MSCs or imDCs, possibly due to enhanced long-term immune tolerance. This “triple therapy” could provide a potentially promising paradigm for clinical islet transplantation to treat T1D.
